# Exploring the implementation of the Community for Successful Ageing (ComSA)program in Singapore: lessons learnt on program delivery for improving BioPsychoSocial health

**DOI:** 10.1186/s12877-019-1271-3

**Published:** 2019-10-30

**Authors:** Su Aw, Gerald C. H. Koh, Chuen Seng Tan, Mee Lian Wong, Hubertus J. M. Vrijhoef, Susana Concordo Harding, Mary Ann B. Geronimo, Zoe J. L. Hildon

**Affiliations:** 1 Saw Swee Hock School of Public Health, National University of Singapore, Tahir Foundation Building, 12 Science Drive 2, #08-01, Singapore, 117549 Singapore; 2 Saw Swee Hock School of Public Health, National University of Singapore, Tahir Foundation Building, 12 Science Drive 2, #10-03G, Singapore, 117549 Singapore; 3 Saw Swee Hock School of Public Health, National University of Singapore, Tahir Foundation Building, 12 Science Drive 2, #10-01, Singapore, 117549 Singapore; 4 Department of Patient and Care, Maastricht University Medical Centre, P.Debyelaan, 25, 6229 HX Masstricht, The Netherlands; 5 Department of Family Medicine, Vrije Universiteit Brussel, Brussel, Belgium; 6 Tsao Foundation, 298 Tiong Bahru Road, #15-01/06 Central Plaza, Singapore, 168730 Singapore; 7 Bloomberg School of Public Health, The John Hopkins University, 111 Market Place, Suite 310, Baltimore, Maryland 21202 USA; 8 London School of Hygiene and Tropical Medicine, Faculty of Public Health and Policy, Department of Global Health and Development, Keppel street, London, WC1E 7HT UK; 9 Saw Swee Hock School of Public Health, National University of Singapore, 117549, Singapore, Singapore

**Keywords:** Delivery system evaluation, Successful ageing, BioPsychoSocial health, Implementation science

## Abstract

**Background:**

The Community for Successful Ageing (ComSA) program has implemented overlapping BioPsychoSocial (BPS) components as part of a Community Development (CD) grassroots and volunteer-led initiative. Implementation of such multi-component programming is influenced by known program characteristics including *novelty*, *complexity* and *observability* as well as related organizational factors. As such, we explored ComSA CD’s implementation from the organizational perspective, seeking to inform program improvements.

**Methods:**

We conducted four focus groups with program staff, partners and trainers (total *N* = 21 participants). Findings were analysed using an interpretative approach and synthesized into a line of argument informing lessons learnt.

**Results:**

An implementation framework was identified. It is guided by considering the influence of known program characteristics across **major themes**, representing three core implementation stages. These and *supporting sub-themes* are elaborated in turn:

1. **Creating commitment toward the program** was challenged by novelty and at times a lack of shared understanding of ComSA CD, particularly relating to the S component. Overall, *cohesion within organizational contexts* and having a *strong rapport with the community* (ability to engage) were needed to persuade volunteers and participants to commit to the program.
2. **Coordination and resource allocation** were influenced by the complexity of interconnecting BPS components - requiring *aligning communication between partners and adapting the BPS sequence,* given *the separated management structure* of program trainers. Efficiency of resource utilization was constrained by the ability to pool and match resources given the *limited manpower* and community partners who worked-in-silo due to a *KPI-centric culture*.
3. **Collaborative program monitoring and appraisal** increased observability of the program’s benefits, but depended on partners’ prior commitment. Despite appreciating its holistic BPS programming, *dropout rate was used as a way to gauge program success,* which has limited interpretability. Occasional uncertainty about the program value contributed to concerns about *duplicating existing ageing programs,* particularly those related to the B component.

**Conclusion:**

Lessons learnt for improving BPS programming include (1) eliciting better participants’ buy-in and shared program vision, (2) increasing adaptability of BPS sequence and building a culture of shared values for working together (3) and developing comprehensive monitoring systems for program appraisal.

## Background

Current empirical and theoretical models on successful ageing emphasize the role of resilience [1–3] and proactivity [4] in coping with health and age-related adversities and maintaining good Quality of Life in old age. To promote resilience and Quality of Life [5, 6], many community programs focus on BioPsychoSocial (BPS) health promotion of community-dwelling older adults before or during the onset of retirement [7–11].

These programming approaches have led to explicit attempts to theorize and test the interconnections among BPS health constructs, and their effect on Quality of Life. Health is conceived as a holistic endeavour in the community development initiative of the wider Community for Successful Ageing program (ComSA CD) in Singapore. The program model theorizes that Biological self-care at older ages can be learnt and maintained. It relates this process to positive ageing perceptions [12] and the psychological ability to appraise one’s life as meaningful and maintain one’s sense of identity, despite facing age-related adversity [1, 3]. Ability to engage in social participation and civic engagement is turn related to psychological and biological health, towards improving the community’s capacity to address needs of other older people [6]. Empirical validation of these theorized BPS interconnections was used to inform the design and sequence of the BPS program components [13].

Yet, regardless of BPS theory, implementing the intended program components in sequence, by multiple community partners, poses its own challenges. The purpose of the current analysis is to explore the organizational perspective on the delivery of ComSA CD, unpacking influences on its implementation and lessons learnt to improve BPS programming for older adults’.

### Factors influencing implementation

Referring to the implementation science literature, there are various stages that people undertake when they implement a new technology, or program. These include those related to planning (resources, outreach strategy of the program), engaging (participants to enrol in the program), executing, and reflecting/evaluating (e.g. about the program benefits and implementation strategy) [14]. How these stages are carried out are often influenced by context - or the complex adaptive system that forms the dynamic environment in which implementation is situated [15].

This consists of the organizational contexts that key implementing actors face, such as the leadership quality, governance and communication systems, manpower, funding, as well as convening power of the organization [14, 16–18]. Organizational contexts are in turn situated within the larger policy, and community setting characterized by the existing degree of partnership, trust, and respect among community organizations.

At the same time, the program’s characteristics [19], such as its *complexity*, *novelty*, and *observability* of relative advantage influence implementation and ultimately it’s successful diffusion. One way of determining complexity is by assessing the number of program components, numbers and levels of outcomes targeted [20]. BPS programs with multiple components to be delivered in a fixed sequence, are more challenging to implement due to the greater level of coordination required.

Novel programs which significantly alter work flow and relationship dynamics between implementers are also likely to require close communication and coordination [21]. On the other hand, observability of a program refers to the degree to which it allows implementers to perceive its benefits and advantage over similar programs [22, 23]. More observable programs are naturally easier to evaluate and reflect upon to sustain commitment.

According to the Normalization Process Theory, implementation consists of a set of feedback loops, and is not a linear process [24]. Implementing organizations can either negotiate organizational contexts according to the needs of the program or instead adapt the program to accommodate the organizational contexts. Appraising both the organizational contexts alongside the program’s characteristics and delivery during program planning and implementation will shape the success of its delivery. This reflexive process therefore helps to determine implementation outcomes ranging from its extent of acceptability and adoption [25], fidelity versus adaptation [26], as well as sustainability.

### Program description

ComSA CD targets six dimensions of health for community-dwelling older adults living in the Singapore neighbourhood estate of Whampoa (2016–2018) through three Biological, Psychological and Social program components and related sub-components [27]. ‘Self-Care on Health of Older Persons’ (SCOPE) targets largely Biological (B) health through 16 weeks of lessons on self-care and related behaviours, including the Bio-Psychological (BP) sub-component on improving the perceptions of ageing [28]. Four self-care behavioural areas are targeted, including healthy eating, exercise, health monitoring and chronic disease management, and communication with health professionals.

‘Guided Autobiography’ (GAB) targets mainly Psychological (P) health in terms of life satisfaction through 8 weeks of structured reminiscence group therapy about life experiences, which has been shown to improve life satisfaction, ego-integrity, sense of mastery, positive well-being and social integration [29, 30]. The Psycho-Social (PS) sub-component of improving interpersonal communication on emotive issues is emphasised.

‘Sharing Wellness and Initiatives Group’ (SWING) targets Social (S) and Socio-Communal (SC) health of older adults in terms of social support and civic engagement. It includes an 8 week participatory workshop to foster critical community assessment and thinking on community solutions, which are known operational domains for community development and capacity-building [31]. More details on the program structure, underpinning theory and content can be found elsewhere [13].

ComSA CD was commissioned in 2014, as part of a City of All Ages Initiative [32] by the Ministerial Committee on Ageing to create age-friendly neighborhoods in Singapore. The neighborhood of Whampoa was selected as a pilot-program site, as 23.5% of its residents were 65 years and above, compared to the average of 11% in other estates [33]. While community programs particularly targeting biological health of older adults have been implemented [34], thus far, none is presented as holistically targeting BPS health, and uses a community development approach [31].

The lead implementer was a non-governmental organization, Tsao Foundation, who worked in partnership with the People’s Association [35] a grassroots organization with links to other community organizations in Whampoa. Partners’ roles include outreach to enrol their members as participants, providing space and operational support to run the classes. Program activities were delivered face-to-face by volunteer trainers, across nine resident centres and one community club in Whampoa under the People’s Association, and at three other community organizations. Volunteer trainers were coached by master trainers from Tsao Foundation. In Singapore, each neighbourhood constituency has one or two community clubs, as well as several resident centres across different precincts termed as ‘resident centre’ zones. These sites offer social activities for residents and are mostly run by volunteers.

### Aims and objectives

Despite the intricacies inherent in implementing multi-component holistic programs for older adults, few studies have explored the implementation challenges of BPS programs. Addressing this gap, we aimed to explore the organizational perspective on the implementation of ComSA CD. The analysis is focused on unpacking program’s implementation contexts and outcomes, to derive a set of lessons learnt for improved BPS programming. Towards our aim, our specific objectives were two-fold:

1. To explore organizational perspectives on the ComSA CD program characteristics (relating to novelty, complexity and observability) alongside implementation experiences and challenges.
2. To derive lessons learnt according to identified challenges and consider how to redress them to positively influence implementation outcomes.

## Methods

### Study backdrop

This study is part of a larger longitudinal mixed-methods evaluation (2016–2018). We have previously elaborated the program’s BPS theory, and validated the association of BPS health with Quality of Life as well as their interconnections [13]. The current study focuses on the program’s implementation, while a mixed-methods outcome evaluation is in preparation.

### Study design

We report a qualitative study exploring organizational perspectives on ComSA CD delivery. We worked closely with program implementers (SHC and MABG) to design the structure, content and composition of Focus Group Discussions (FGDs).

### Data collection

We conducted four FGDs lasting 1 to 2 h in English. These were held at the Whampoa resident centre, community club and Tsao Foundation, with (A) program staff/ trainers, and (B) community partners (see Table 1).

**Table 1:** Focus Group Discussion Sampling Characteristics (*n* = 21)

Sampling categories	N^o^ and type of FGD	N^o^ of participants	Gender (F: M)
(A) Program staff and trainers	1st Group: GAB/SCOPE trainers	*n* = 8	7:1
	2nd Group: Senior SCOPE/SWING trainers, participant volunteers and operational staff from Tsao Foundation	*n* = 5	4:1
(B) Community partners	3rd Group: Resident Centre (RC) managers	*n* = 5	4:1
	4th Group: Senior Managers from Whampoa Community Club Management Committee (CCMC)	*n* = 3	0:3
Total		*n* = 21	15: 6

Using the attendance list of each class from Tsao Foundation, we recruited eight trainers in FGD1, consisting of four B program component and four P program component trainers. Half of each had delivered classes with the highest participant dropout and the other with the lowest dropout. In FDG2, in order to contrast perspectives directly, we mixed five senior trainers, participant volunteers and operational staff from Tsao Foundation. In FGD3 we talked to five volunteers who managed the day-to-day operations of the Resident Centers. Lastly, in FGD4 we involved three senior management staff from the Whampoa Community Club Management Committee (CCMC) [36] of the People’s Association, as they were key implementation partners. All participants were recruited via electronic mail, and provided informed consent before participating in the FGDs.

All FGDs were run by SA, who also transcribed the audio-recording verbatim. SA worked with a team of observers who recorded meeting dynamics (ZH and part-time support staff). The qualitative team was female, from multidisciplinary backgrounds (sociology, psychology and public health) with training in qualitative research. Most community partners were recruited during the formative program assessment stage and were known to the researchers, allowing a greater rapport to be established. Nevertheless, the independent role of the research team was emphasized at the start of the FGDs.

The sessions aimed to elicit organizational perspectives on the ComSA CD program characteristics alongside implementation experiences and challenges. FGD topics included: (1) how partners initially perceived and came to adopt the program according to organizational setting and priorities (focusing on program novelty); (2) following program adoption, how partners worked together to implement and adapt the program according to challenges faced and strategies that worked (focusing on program complexity); relating these to (3) how they tracked and appraised the program (focusing on observed benefits).

### Data analysis

Qualitative data were analysed using an interpretative approach. In the first stage, data were organized in NVIVO 11 by filing them by topics listed above. After which, thematic analysis within each topic was carried out, clustering top-line emergent dominant themes (in **bold**), representing stages of implementation. These were then used to anchor and elaborate related subthemes (reported in *italics* at first mention).

**Fig. 1:**
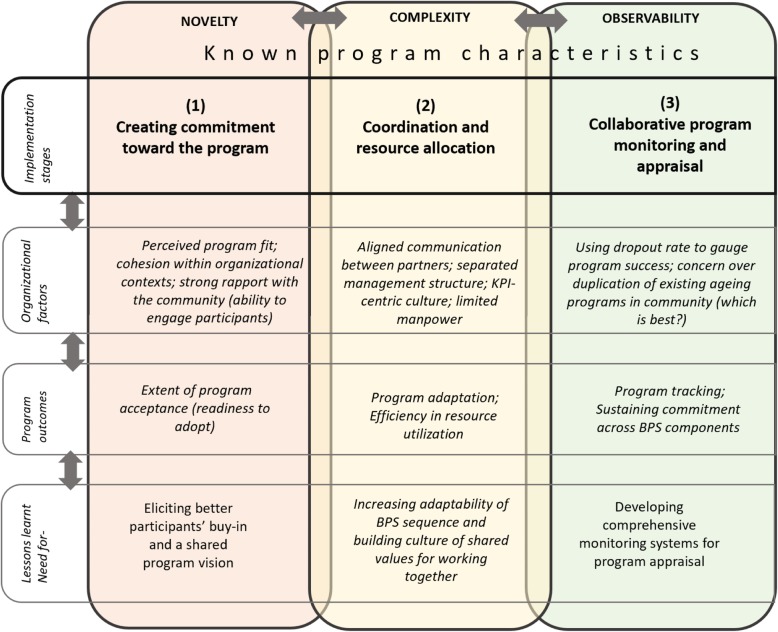
This figure shows a framework structured around known program characteristics and observed feedback loops and anchored onto three core stages of implementation. These are mapped to organizational factors influencing implementation, related outcomes and lessons learnt for each stage of program delivery

In the third stage, interconnections of themes under each topic were explored, generating a line of argument on how the program characteristics, organizational contexts, implementation stages and outcomes were aligned (Fig. 1). Data were analysed by SA and explored with senior qualitative analyst ZH to achieve consensus on the interpretation of the data. Member checks were conducted through the presentation of findings to partner organizations. Saturation was judged to have occurred at the top level of thematic coding by the fourth FGD. Consistency was achieved across participants groups, and both minority voices and wider - collective themes - were accounted for.

## Results

Figure 1 presents the results of our interpretive thematic analysis. The analysis identified three core implementation stages that anchor experiences of organizational contexts and mechanisms, or factors that hinder or promote implementation. The stages are (1) **creating commitment toward the program**, (2) **coordination and resource allocation**, and (3) **collaborative program appraising and monitoring**. We discuss each of these dominant themes in turn, in line with qualifying sub-themes and how they relate to identified program characteristics (novelty, complexity and observability), and ultimately map to implementation outcomes and lessons learnt.

### Creating commitment toward the program

This theme refers to the initial stage of the implementation process, which relies on efforts of partners to create buy-in and persuade their staff to accept the program as a legitimate part of their work. Buy-in from partner organizations was necessary so centre managers could advocate for the program and recruit older people from their centres; likewise, for program trainers so they could persuade participants about the program’s benefits. However, due to the complexity of its multiple BPS components, and in particular the relative novelty of the S program component SWING, it was at times challenging to understand how the program was to be operationalized and coordinated. This influenced the *perceived program fit,* and ultimately its acceptability. *Cohesion within partner organizations,* as well as strong *rapport with the community* (*ability to engage participants)* were needed to create commitment to the program.

The *perceived program fit* of ComSA CD was shaped by its alignment with partners’ organizational goals as well as how well partners understood the program. Due to the *novelty of the* S component SWING, many communal initiatives implemented by SWING members were contested and had to be renegotiated with grassroots leaders who were unfamiliar with participatory community approaches. One such battle related to whether the S community groups participants could represent themselves as members from a specific Resident Centre zone and petition governmental agencies directly on selected community issues without going through official feedback channels of the CCMC.

Unlike other countries, in Singapore, most grassroots bodies are under the purview of the People’s Association, a statutory governing board established in 1960 to manage citizen feedback and promote social cohesion. Grassroots leaders were therefore concerned whether doing so could undermine their role in promoting social cohesion, and ‘flood the inboxes’ of governmental agencies (FGD4, CCMC manager). As partners clarified their intention and collectively specified the boundaries for collective action, it became apparent that the initiatives in the S component SWING could complement the grassroots leaders’ role - increasing trust and buy-in for the program. In turn, this collective operationalization and support from the grassroots leaders for the elder-led action increased trainers’ understanding, acceptance, and resulting confidence, because ‘if they do not understand [the program strategy], they cannot sell it to people. And people cannot rally’ (FGD2, program staff).

Regardless of buy-in from leaders, a strong sense of *cohesion within partners’ organizations* – which can be described as a genuine sense of all ‘pulling together’ to serve and support beneficiaries –was needed so that centre mangers themselves were ‘willing to work’. This was especially important in the absence of formal accountability since most partners depended on volunteer-led support. This sense of cohesion was suggested to be lacking in certain partner organizations, as commitment from the leaders did not necessarily trickle down to the centre managers (FGD3, RC manager). Less committed centre managers were described as providing perfunctory support - ‘reducing themselves to locking and unlocking the centre’ for trainers while others even had problems doing so on time (FGD2, program staff).

Following which, having *strong rapport with the community* was viewed as necessary to persuade older people to commit to the program, especially since most naturally liked being around familiar faces in the community. Centre managers who were well-liked and trusted by older people, were inherently more effective when they advocated for the program. For example, their ‘personal magnetism’ could inspire ‘giving face’ or using their social standing to motivate participation as described– ‘I can just tell them “you all know me, right? So, we’re going to continue with another program now.” They will give me face and come’ (FGD2, program staff).

As centre managers were local figures who had often established rapport with residents, trainers described how their support was important as they entered the community for the first time – ‘because participants look up to them not us, by the time they know us, half the program is done’ (FGD2, program trainer). Simple acts of appreciation, such as ushering and thanking participants for coming, to providing personal reminders to return each week, were powerful tools in boosting attendance. As one trainer explained: ‘when the centre manager wheels the wheelchair-bound lady into class, participants see this man doing this for this lady, they dare not skip class’ (FGD2, program trainer).

Where centre managers went beyond operational support to elicit buy-in and advocate for the program, this optimized delivery conditions for the trainers. Their physical presence helped extend trust to trainers, boosting trainers’ confidence and ability to build rapport with participants. This also enabled trainers to promote group cohesion in class, a factor described as key in retaining participation across B, P and S program components.

In summary, overall, creating a genuine commitment to the program was seen as the most important influencer on outcomes relating to the *extent of program acceptance (readiness to adopt the program).* Lessons learnt are therefore connected to *eliciting better participants’ buy-in and a shared program vision*. Related strategies for this are proposed in the discussion section.

### Coordination and resource allocation

Delivering the multiple components of the BPS program across the 13 community sites required a considerable amount of coordination and resource allocation. Required resources included manpower support from centre managers, program trainers, space as well as technical equipment. Herein, the program’s complexity meant that partners had to work more closely in *aligning communication* to coordinate the sequence of BPS components. However, constraints faced by the *separated management structure* of program trainers necessitated a degree of *program adaptation.* On the other hand, *limited manpower resources* and *KPI-centric organizational culture* influenced the ability to pool and match resources, and therefore the *efficiency of resource utilization*.

One important aspect complicating the delivery of BPS components, was how the B, P and S components were intended to be experienced sequentially, and also that they required quite different modes of facilitation. For example, compared to teaching B self-care, P guided auto-biographical (GAB) needed a less directive approach, while S activities required more group facilitation to galvanize collective action. Facilitators adept at one aspect of program delivery, would not necessarily be so for another, and trainers were not always aware of the mental switch needed when moving to another program component – ‘I think in the train-the-trainer session, it didn’t cover ‘look your mentality needs to be like this for doing SCOPE [B classroom teaching]; but in SWING [S community-led activities], your mentality has to change’ (FGD2, program staff).

Despite regular and constructive meetings, *aligning communication between partners* to ensure the operational demands and timeline of each BPS component complimented existing work schedules was at times challenging. This was particularly relevant for SWING, not only because it was intended to be delivered last, but also because it was a relatively novel component that required experimentation. However, due to the limited volunteer support, partners found it difficult to support the program activities if they were not communicated ahead of time, and their operational demands properly understood. This may explain why one partner perceived having to support the program in a manner likened to ‘filling in the stones as they walk’ (FGD4, CCMC manager). Moving from the pilot-experimental phase, shared web-based scheduling systems, may be needed to improve communication in the scale up of the program.

Another barrier was the *separated management structure* of program trainers, or how the B, and S component trainers were managed by different teams within Tsao Foundation from the P component trainers. Those running the P had additional work roles beyond delivery of guided auto-biographical sessions. This made it harder to coordinate their schedules with B trainers, as well as increase delivery of the P sessions within a short notice. Since the overarching aim was community empowerment, it was seen as optimal to use the B self-care and P guided auto-biography as a platform to build social support before participating in community development initiatives in the S component SWING. Retaining this core element of the program theory while accounting for organizational constraints influenced program adaptation into two tracks (B component SCOPE followed by S component SWING and P component GAB, followed by S component SWING).

Related to efficiency in resource utilization was the ability to pool and appropriately allocate resources. One way of pooling resources was to bring together participants from different community sites and run combined classes. A larger class size in the B or P program components allowed for a higher trainer-to-participant ratio and also guaranteed sufficient numbers to open a new S group after eventual dropout. However, due to a *KPI-centric organizational culture*, it was highlighted that doing so created ‘turf issues’ among some centre mangers as they were judged on the number of participants attending their sites (FGD2, program staff). Having a more participant centred mind-set – as was common in more cohesive organisations – helped counter this focus on KPI and ‘working-in-silo’ setup as described: ‘centre manager whose mind-set is really on the senior will be ok with combining and allowing the senior to decide’ (FGD2, program staff).

Given the multi-ethnic setting (e.g. Malay, Chinese, English etc.) in Singapore, allocating trainers to participants with a similar cultural profile naturally increased their competence in engaging older people. This was at times difficult due to the *limited manpower*. For example, allocating a non-Malay trainer who did not speak fluent Malay to teach B classes due to the lack of a Malay trainer. The resulting mismatch was seen to compromise cultural competency in understanding ‘what makes them (Malay’s elders) tick’ (FGD2, program staff) and motivating B self-care.

In sum, coordination efforts were defied by the complexity of the BPS program, which was somehow aggravated by more novel and unfamiliar aspects of programming, as described earlier. Outcomes relating to *program adaptation and efficiency in resource utilization* were qualified by partners’ abilities to come together and practically share the responsibilities and resources for the program*.* Lessons learnt are consequently connected to *increasing adaptability of BPS sequence and building culture of shared values for working together*. Related strategies for this are discussed in the discussion section.

### Collaborative program monitoring and appraisal

Sustaining partners’ efforts at coordination and commitment across the program’s BPS components, depended by how they monitored and appraised the benefits of the program and their partnership. Engaging in collaborative program monitoring and appraisal was positioned as a feedback loop, connecting back to the level of prior program commitment from partners. Committed centre managers were more likely to be physically present during the program, where they could observe participants and appraise changes, albeit in a subjective way as described: ‘There is one grandma who is very low-spirited. After coming to the course, she is now better’ (FGD3, RC manager) This contrasted with less committed centre managers who ‘either don’t turn up (in the program), or just show up for a while’ (FGD2, program trainer).

The lack of a formally agreed and comprehensive monitoring system among partners was identified. In its absence, *dropout rate* was used as a common but misleading gauge of program progress. Attrition is a known phenomenon in the early stages of recruitment, as one participant noted: ‘One trend is that we have seen lots of attrition [ …] if they manage to get 10 on the first day, then on the second day we might see 5 or 4 or 3. Then, over time, we may only see 4 or 5 only. But some actually told us that this is the normal attrition rate for programs [ …] So I’m not sure about how beneficial it is [using this barometer]’ (FGD4, CCMC manager).

Although partners appreciated the program’s overall BPS structure, its advantage over other ageing programs still sometimes needed ‘selling’ to certain centre managers, who were concerned about *duplication of ageing programs in the community*, particularly that of B health program component. One challenge was comparing and deciding which B program to advocate to residents: ‘how can we ascertain the value-added-ness of a program? [ …] How can we tell that SCOPE is above the Health Promotion Board or Ministry of Health’s programs?’ (FGD4, CCMC manager).

Monitoring and appraising the program’s effects were inherently related to *sustaining commitment across BPS components.* An obvious lesson learnt from these analyses points to the need for *Developing comprehensive monitoring systems for program appraisal* that can be shared and collected in an objective and standardized way. These should include implementation indicators aligned to the key types of outcomes identified herein.

## Discussion

In this study, we illustrated how the implementation of ComSA CD was an overlapping process, consisting of a set of feedback loops, and therefore not a linear process [24]. The key implementers involved attempted to negotiate organizational contexts according to the program’s needs while adapting the program as it unfolded. The stages of implementation identified in our framework share conceptual similarities with implementation processes proposed by Carl May [37]. For example, the initial stage of creating commitment to the program requires making sense of the program and cognitive participation (to legitimize and persuade others to enroll). This is followed by collective action and reflexive monitoring of the program.

However, one advantage of our implementation framework is the explicit mapping of how different organizational contexts influences different stages of implementation, as well as implementation outcomes arising from each stage. Nevertheless, organizational contexts highlighted in the framework are not meant to be exhaustive. As ComSA CD is driven by voluntary community partnerships, organizational factors influencing its implementation, will likely differ from those influencing the formal, contractually-bound partnerships of corporate organizations.

### Lessons learnt for BPS programming

#### Creating commitment toward the program

One key finding from this study was that community partner’s’ convening power, and skills to elicit buy-in from residents was crucial to boost program participation [17]. However, this varied from partner to partner, depending on the personal magnetism, commitment, and management of their volunteers such that they were cohesive and ‘willing to work’. To overcome resource limitations, new ways to harness participants as program champions in the community may be required. Programs can also consider enlisting the help of public figures and opinion leaders (e.g. celebrities, charismatic program staff) to help promote the program.

Novel programs such as ComSA CD are likely to significantly affect work flow and power dynamics between implementers. For example, in galvanizing participants and offering them another platform for action on community issues, the S component SWING was initially seen as contesting the role of grassroots leaders from the People’s Association in managing residents’ feedback. Brainstorming the program’s vision and strategy together, was therefore necessary to build trust, and ease changes to the working dynamics; most notably moving from curation to facilitation of collective action.

#### Resource coordination and allocation

While our BPS theory suggested that the ideal program flow was to sequentially deliver the B-P-S components, one barrier faced was the separated management of trainers. This is especially relevant for ‘tightly coupled’ programs such as ComSA CD, or multiple-component programs with a fixed sequence. Such program often require more coordination [24], communication, and adaptation to different organizational contexts [26, 38]. A more flexible program sequence was therefore necessary to ease coordination.

Despite adaption of the program flow, individual empowerment and bonding through smaller groups in the B and P components prior to S SWING facilitated the overarching aim of community development and maintained program integrity. Following which, identifying core implementation techniques that make each BPS component work is key to improving and replicating the program.

Similar to other studies, we found that aligning partners’ interests, and managing conflict and turf issues were key challenges faced in community health partnerships [18, 39]. Overcoming partners’ KPI-centric organizational culture and focusing on participants’ experience to drive resource coordination/allocation was not always possible, given differences on why and how community activities should be organized.

Another key lesson learnt thus refers to the importance of building cohesion and shared values among partners before implementation. Studies have shown how internalization of norms, through leadership and socialization, are critical to governing, ensuring accountability and resolving disputes in voluntary community partnerships [18, 40]. Leaders from a larger program committee can help to neutralize territorial anxieties, by identifying a common vision and win-win opportunities, and building a shared participant-centered culture. To increase sharing of ideas, resources and power, boundary-spanning leaders who can bridge partners’ perspectives and cultures may be more effective compared to leaders with a narrow range of expertise [16, 41].

#### Collaborative program monitoring and appraisal

An emergent lesson learnt was the need for a more comprehensive monitoring system to appraise the program benefits and unique value, in relation to other ageing programs in the community. This was needed for ComSA CD, given the many B programs implemented in the community as part of Singapore’s Successful Ageing Action Plan.

Evaluation checklists could serve as a useful tool and communication aid for partners to reflect on program effects, their readiness for program adoption and implementation. Embedding these checklists within quality improvement cycles, to monitor, plan and innovate new joint improvement goals is likely to foster a spirit of learning and experimentation. Furthermore, coordination could be vastly improved by the introduction of shared information management systems such as web-based team calendars for scheduling or decision support tools [42].

#### Across implementation stages

Regular communication to collectively specify and re-specify the program’s meaning and theory was central in creating perceptions of fit and trust in the program [37]. This was challenging due to ComSA CD’s complexity. What was needed - and often harnessed - was to communicate the overall agreed program structure, while allowing continuous learning of what works best to emerge. This was particularly important for the S community development component. This component of the program adopted a relatively novel participatory approach that was less intuitive in Singapore’s top-down, government-led grassroots context [43, 44].

#### Strengths and limitations

Due to time and resource constraints, not all community partners were recruited to the FGDs, resulting in a small but targeted sample size. We did however account for the breadth of partners’ perspectives where possible. For example, while we were not able to recruit Malay community partners, we recruited program trainers and centre managers who were in charge of the Malay classes instead. To ensure the validity of the results, we conducted member checks by presenting findings to program staff and partner organizations. Lessons learnt identified on improving BPS programming across the different implementation stages can be transferred across multi-component programs driven by voluntary community partnerships.

## Conclusion

Organizational factors played a key role in influencing trainers’ ability to elicit change among participants on the ground. Further research testing the optimal sequence (B component SCOPE followed by S component SWING versus P component GAB followed by S component SWING) and the magnitude of change on BPS and Quality of Life outcomes are needed. Such research will enable policymakers and implementers to unpack the ‘black box’ of complex, multi-component BPS health promotion programs, towards successful ageing in the community.

## Data Availability

The datasets used and/or analyzed during the current study are available from the corresponding author on reasonable request.

## Abbreviations

B: Biological
BP: Bio-Psychological
BPS: BioPsychoSocial
ComSA: Community for Successful Ageing
FGD: Focus Group Discussions
GAB: Guided Autobiography
NPT: Normalization Process Theory
P: Psychological
PS: Psycho-Social
S: Social
SC: Socio-Communal
SCOPE: Self Care on Health of the Older Persons
SWING: Sharing Wellness and Initiatives Group
